# Diet and Skin Cancer: The Potential Role of Dietary Antioxidants in Nonmelanoma Skin Cancer Prevention

**DOI:** 10.1155/2015/893149

**Published:** 2015-10-25

**Authors:** Rajani Katta, Danielle Nicole Brown

**Affiliations:** ^1^Department of Dermatology, Baylor College of Medicine, 1977 Butler Boulevard, Suite E6.200, Houston, TX 77030, USA; ^2^Department of Dermatology, Baylor College of Medicine, Houston, TX 77030, USA

## Abstract

Nonmelanoma skin cancer (NMSC) is the most common cancer among Americans. Ultraviolet (UV) radiation exposure is the major risk factor for the development of NMSC. Dietary AOs may prevent free radical-mediated DNA damage and tumorigenesis secondary to UV radiation. Numerous laboratory studies have found that certain dietary AOs show significant promise in skin cancer prevention. These results have been substantiated by animal studies. In human studies, researchers have evaluated both oral AO supplements and dietary intake of AOs via whole foods. In this review, we provide an overview of the role of AOs in preventing tumorigenesis and outline four targeted dietary AOs. We review the results of research evaluating oral AOs supplements as compared to dietary AOs intake via whole foods. While these specific supplements have not shown efficacy, intake of AOs via consumption of whole foods has shown some promise. Lessons learned from the field of hypertension research may provide important guidance in future study design. Further research on the role of dietary AOs in the prevention of NMSC is warranted and should focus on intake via whole food consumption.

## 1. Introduction

Nonmelanoma skin cancer (NMSC) is the most common cancer among Americans. The number of cases of NMSC, which includes basal cell carcinoma (BCC) and squamous cell carcinoma (SCC), exceeds that of breast, lung, prostate, and colon cancer combined. Ultraviolet (UV) exposure is the major risk factor for the development of skin cancer, and while public health campaigns have been somewhat successful in modifying the behaviors that increase UV exposure, there is still significant exposure that occurs through intentional tanning, use of tanning beds, and incidental exposure. Researchers have therefore studied other avenues of skin cancer prevention, including dietary modification through the intake of antioxidants (AOs).

In this review, we provide an overview of the role of dietary AOs in preventing tumorigenesis. Laboratory and animal studies have outlined potential mechanisms of action and have shown promise. There have been a limited number of large, longer-term human studies, and these have evaluated four AOs in depth. Researchers have also begun to evaluate the dietary intake of AOs via whole foods. While these specific supplements have not shown efficacy, intake of AOs via consumption of whole foods has shown some promise. Lessons learned from the field of hypertension research may provide important guidance in future study design. Further research on the role of dietary AOs in the prevention of NMSC is warranted. While such research may include evaluation of other supplements, or combinations of supplements, it must include further evaluation of intake of dietary AOs via whole food consumption.

## 2. Antioxidants and Their Role in Photocarcinogenesis

NMSC tumorigenesis is an extended, multistage process, consisting of initiation, promotion, and progression. Damage from free radicals is known to play a role in the initiation of this process [1]. UV radiation and exposure to environmental pollution generate free radicals. Both UVA and UVB radiation induce DNA damage; however, UVA radiation is more associated with free radical-mediated damage [1].

Free radicals are molecules that contain unpaired electrons and induce direct oxidative damage to proteins, lipids, and DNA. Most free radicals in the body exist in the form of reactive oxygen species (ROS) [1]. ROS are known to damage the bases and deoxyribosyl backbone of DNA [2]. More specifically, free radicals (mainly as singlet oxygen or hydroxyl radicals) damage DNA through the formation of oxidized pyrimidine bases and single strand DNA breaks [3]. This DNA damage may lead to tumorigenesis.

Free radicals damage not only DNA, but also cellular proteins and lipids. Direct oxidation of enzymatic proteins leads to activation of pathways that produce new proteins. These processes can increase cell proliferation and inflammation [4]. Free radical-mediated peroxidation of lipids promotes destruction of the cell phospholipid bilayer. Through these mechanisms, the accumulation of oxidative stress has been found to promote apoptosis [5].

Furthermore, UV radiation can lead to immunosuppression, hampering the ability of immune cells to recognize and combat cancer cells. As tumorigenesis progresses, other biochemical changes can result in increased angiogenesis and capacity for tumor invasion [6].

AOs combat these processes. They work through a number of mechanisms that prevent these oxidative reactions and subsequent DNA and cellular damage. Some have also been shown to act through upregulation of genes encoding for enzymes, which are capable of neutralizing ROS [7]. There are many naturally present AOs in the skin, and there exists a decreasing concentration gradient of these substances from the epidermis to the dermis [8]. These innate skin AOs include enzymes such as superoxide dismutase and glutathione peroxidase, as well as nonenzymatic substances such as vitamin C and vitamin E [1, 9].

While the body has mechanisms in place to neutralize ROS, accumulative oxidative stress from UV exposure can inundate these mechanisms. Therefore, researchers have turned to exogenous AOs. Preliminary studies in humans have shown that individuals with BCC have higher serum markers of oxidative stress and lower serum levels of dietary AOs [10]. Therefore, dietary AOs have been evaluated for their potential efficacy at reducing UVA-induced photocarcinogenesis.

Multiple animal studies, some ranging back decades, have found that AOs provide protection against skin cancer. Some have focused on supplementation with single AOs, while others have focused on varying combinations. In hairless mice exposed to UV light, a significant reduction in incidence of malignant and precancerous lesions was seen in mice that had received supplemental vitamin C in the diet [11]. In another study of mice exposed to a topical carcinogen, supplementation with beta-carotene reduced the number of tumors by 32%, while vitamin E supplementation reduced number of tumors by 25% [12]. In another study, selenium supplementation in the diet prior and during UV irradiation of mice was shown to provide significant dose-dependent protection against skin cancer [13].

In another study of hairless mice exposed to UV radiation, 30% of the mice fed a regular diet developed frank SCC, while only 7% of those fed a special diet developed SCCs [14]. This diet included a mixture of vitamins C and E with glutathione and butylated hydroxytoluene. In a later study of mice treated with a potent carcinogen, a nutrient mixture added to their diet significantly inhibited the incidence and multiplicity of skin tumors. This mixture included vitamin C, selenium, green tea extract, and other naturally occurring AOs [15].

Laboratory studies and further animal studies have identified potential mechanisms of action for these effects. It is important to note that the beneficial effects noted in these studies may be dependent on other factors. In fact, researchers write that “under certain conditions both water soluble antioxidants (e.g. vitamin C and urate) and the lipid soluble antioxidant tocopherol (vitamin E), promote or even induce peroxidation [16].” For example,* in vitro* studies have found that in mild oxidative states and in the absence of other co-AOs such as vitamin C, vitamin E may act as a prooxidant [17]. This mechanism, and the associated potential for adverse effects of AOs, is further described in the later section detailing the biochemical process of oxidation.

While there is clear benefit in multiple laboratory and animal studies, studies performed in human subjects have provided conflicting results. While there are a large number of identified AOs, this review focuses on four specific AOs for which longer-term human studies have been performed: vitamins C and E, beta-carotene, and selenium [18].

### 2.1. Vitamin C

Vitamin C, or ascorbic acid, is a water-soluble vitamin present most abundantly in fruits and vegetables. It serves as a cofactor of multiple different enzymes in the human body including prolyl and lysyl hydroxylase [7]. These enzymes are essential for the synthesis, cross-linkage, and stability of collagen. Vitamin C also serves as an intracellular antioxidant, and in studies it has been shown to provide protection against UV radiation and carcinogenesis.

In a study of cultured keratinocytes, researchers found that vitamins C and E counteracted the increase in ROS induced by acute UVB irradiation, and in combination protected against UVB-induced apoptosis [19]. In normal human oral keratinocytes, researchers compared the protective roles of vitamins C and E in oxidative stress imposed by smokeless tobacco. Vitamins C and E, alone and in combination, offered significant protection [20]. Vitamin C also impacts DNA repair. In a study of human dermal fibroblasts treated with vitamin C, researchers found an increased expression of genes associated with DNA replication and repair, and the fibroblasts demonstrated faster repair of oxidatively damaged DNA bases [21].

### 2.2. Vitamin E

Vitamin E differs from the other AOs reviewed here in that it actually represents a group of closely related molecules. These 8 different molecules include 4 tocotrienols and 4 tocopherols [22]. These fat-soluble substances are found in foods such as soybeans and wheat germ, and the naturally occurring form D-alpha tocopherol has the greatest biological activity. When synthesized, however, it forms together with l-alpha tocopherol, and this l-isomer has less biological activity. Therefore, when referring to vitamin E, the international unit (IU) designation is utilized and refers to the same level of biological activity, regardless of the form of vitamin E utilized.

Vitamin E is lipid soluble and has been shown to prevent membrane lipid peroxidation by ROS. In a study of mouse keratinocytes, vitamin E treatment prior to UVB radiation was able to reduce the UVB-associated epidermal damage [23]. In human fibroblasts exposed to UVA light, vitamins C and E showed photoprotective potential [24].

### 2.3. Carotenoids

Carotenoids are a group of plant compounds which impart a bright color to the different fruits and vegetables in which they are found, such as carrots, squash, and sweet potatoes. There are hundreds of carotenoids, with about 40 said to be present in the typical human diet [25]. Beta-carotene stands as the most studied, since in the majority of countries it is the most common carotenoid consumed.

In laboratory and animal studies, carotenoids have been shown to impact carcinogenesis, with several postulated mechanisms. Carotenoids can be converted by the body to retinoids, which have suppressed carcinogenesis in multiple animal tumor models [26].

Another mechanism focuses on the AO capabilities of carotenoids, which have the ability to quench singlet oxygen and scavenge free radicals. In a study of cells from a human liver cell line, carotenoids provided protection against oxidant-induced lipid peroxidation [27]. Of note, this protection was found to be independent of any proretinoid activity [28]. In animal studies, beta-carotene has suppressed lipid peroxidation [29, 30].

### 2.4. Selenium

Selenium is a trace mineral and is found in different food sources, including plants grown in soil with high selenium concentrations, as well as some meats, fish, and other sources. Selenoproteins are proteins that contain selenium in the form of an amino acid. In knockout mice studies, mice lacking selenoproteins in keratinocytes developed skin abnormalities, and it was found that selenoproteins are essential AOs which play an important role in keratinocyte growth and viability [31].

In laboratory studies, selenium has demonstrated effects against carcinogenesis. Selenium derivatives have induced apoptosis in different human tumour-derived cell lines, including skin cancer [32]. In a mouse carcinogenesis model, a selenium compound significantly reduced preneoplastic skin lesions, with significant decrease in cell proliferation and significant enhancement of apoptosis [33]. In the same carcinogenesis model, treatment with selenium also resulted in inhibition of lipid peroxidation in skin, as well as elevation of AO enzymes, including catalase and superoxide dismutase [34].

## 3. Human Subject Studies: Antioxidant Supplements

There are four AO supplements for which large, longer-term human research studies are available. Studies of these four supplements in humans have not supported their role in skin cancer prevention. Randomized controlled trials (RCT) of beta-carotene, selenium, and combination supplements (including various combinations of vitamin C, vitamin E, beta-carotene, selenium, and other substances) have not been shown to reduce the incidence of NMSC in men or women [35–41]. Details of these studies are provided in Table 1.

## 4. Human Subject Studies: Estimated Dietary Intake via Supplements Combined with Food 

Studies estimating dietary intake of AOs via the combination of supplements and whole foods have not shown promise in reducing the incidence of NMSC (Table 2). However, these studies have considered the intake of AOs to be simply additive. Research supports the idea that AOs provided in the form of isolated supplements function in a different manner than that provided in the form of whole foods. Therefore, it would be ideal to differentiate between these forms of AO intake.

## 5. Human Subject Studies: Serum Levels

At this time, there are limited studies evaluating serum levels of these specific AOs in NMSC prevention (Table 3). The largest of these studies is a cohort study of 485 adults.

Conflicting results have been seen in these small studies. In addition, the issue of timing when performing serum studies is an important one. While in one study lower than mean selenium levels were associated with skin cancer, it was noted that neoplastic tissue sequesters selenium [42]. This may then lower serum levels, thus magnifying the importance of timing when performing serum studies.

In addition, it is not known how well serum AO levels reflect dietary intake. In the case of serum cholesterol, for example, it is well known that genetic differences can result in markedly different serum cholesterol levels despite the same level of dietary intake.

## 6. Human Subject Studies: Antioxidant Intake via Whole Foods

Designing a study to evaluate the effects of AO intake via whole foods is very challenging. One small, well-designed dietary intervention trial showed promise [43]. While the intervention did result in increased intake of vitamin C, beta-carotene, and fiber, the study was focused on the effects of a low-fat diet. A larger experimental study also looked at the effects of a low-fat diet. The subjects were given a dietary plan which entailed decreasing fat intake to less than 20% of caloric intake and consuming at least 5 servings of fruits and vegetables daily [44]. This study did not demonstrate efficacy, but the study design must be considered in evaluating the results. A separate prospective observational study did not focus on macronutrients (i.e., fat), but rather focused on the “combined consumption of foods,” and it did find that a “vegetable and fruit” pattern decreased NMSC as opposed to a “meat and fat” pattern [45]. Details of these studies are shown in Table 4.

## 7. The Biochemical Process of Antioxidation: A Proposed Explanation for the Lack of Efficacy of Supplements

Given the results of large-scale, randomized, placebo-controlled trials, we conclude that these 4 AO supplements, in the reported doses and for the studied duration of intake, are not effective tools for skin cancer prevention. On the other hand, while analysis of dietary intake of AOs via whole foods has not provided clear conclusions, some promising trends have emerged.

One reason for this reported difference in efficacy may be due to the biochemistry of AOs and their downstream effects. The process of oxidation, and correspondingly antioxidation, is not a straightforward, single-step process, nor is it a straightforward chain of events. It represents instead a finely balanced system, as has been shown in studies of beta-carotene and lung cancer.

In an interventional study, male smokers treated with beta-carotene supplements over 5–8 years had a higher incidence of lung cancer [46]. A later study of smokers also showed an increase in lung cancer among subjects taking beta-carotene supplements with vitamin A supplements [47]. Animal studies helped to identify a possible explanation. In a study of ferrets exposed to smoke, markedly different effects were seen with low dose of beta-carotene supplementation (corresponding to dietary intake) versus high dose intake (corresponding to pharmacologic doses). With high doses of beta-carotene, increased cell proliferation was seen [48].

The biochemistry of this process provides an explanation. Free radicals can cause damage due to the presence of an unpaired electron. AOs such as beta-carotene may neutralize free radicals by providing an electron of their own. In the process, though, the AO itself now contains an unpaired electron. Vitamin C can act to neutralize this newly created prooxidant. Smokers, who are known to have a lower intake of vitamin C, would therefore be at higher risk from isolated beta-carotene supplements.

Further animal studies have shown that the explanation is likely to be even more complex. In cases of a limited (as opposed to a well-balanced) diet, beta-carotene supplements have resulted in significant exacerbation of UV carcinogenesis. The researchers explained that, when beta-carotene exerts its AO effects, the newly created beta-carotene radical cation contains an unpaired electron and is therefore strongly oxidizing. The researchers concluded that “the photoprotective effect of beta-carotene reported earlier by others…might depend on interaction with other dietary factors that are either absent, or present in ineffectual concentrations, in the semi-defined diet in which exacerbation of UV carcinogenesis occurs. Those factors could be other carotenoids, their isomers, or some yet unidentified phytochemical(s) [43].”

A related conclusion was drawn by Chang et al. While their meta-analysis of 10 large RCTs with vitamins and supplements known to have AO properties showed no reduction of NMSC incidence, the researchers did draw a distinction between supplements and whole foods [41]. They stated that when consumed in a fruit or vegetable, these AOs are consumed in relative quantities with one another that may be vital to their AO qualities.

## 8. Directions for Future Study: The Need to Study Whole Foods

Large RCTs of 4 specific AO supplements have not shown efficacy. While research is underway on the potential of other AO supplements, and combinations of AO supplements, it is imperative that future studies also target interventions that integrate dietary AOs in the form of whole foods. In this respect, much can be learned from accomplishments made in the area of diet and hypertension control. Hypertension researchers have had limited results with isolated dietary supplementation but have had success through interventions of whole foods, specifically the Dietary Approaches to Stop Hypertension (DASH) diet.

Prior to whole food investigations, one study showed that nutritional supplementation with calcium, magnesium, potassium, or fish oil had no effect on reducing blood pressure [49]. Another study found the effects from calcium supplementation alone to be too small to be clinically significant [50]. Furthermore, magnesium supplementation had equivocal results [51].

After concluding that dietary supplements lacked promise, a RCT of the DASH diet, a diet rich in fruits and vegetables and low in fat, was conducted. This diet significantly reduced both systolic and diastolic blood pressure in US adults when compared to a typical American diet [52]. Further studies have shown that the DASH diet in combination with a diet low in sodium is the most effective dietary intervention for reducing blood pressure [53].

A major reason to study whole foods is that the nutrients present in supplements are by necessity limited to those that researchers have thus far isolated, identified, and determined to be most worthy of study. As further research in the area of phytonutrients has progressed, researchers have discovered many other potential protective plant compounds.

*In vitro* studies have shown that a number of other phytochemicals may have significant AO effects. Studies have found that compounds such as grape seed extract, resveratrol [from grapes], and ellagic acid [found in foods such as raspberries] are potent scavengers of superoxide radicals and that these compounds are able to protect cells from hydrogen peroxide-induced DNA damage [54]. Other laboratory and animal studies have supported the effects of multiple other phytonutrients, including curcumin (found in the spice turmeric), lycopene (found in tomatoes), and genistein (found in soy) [55, 56].

Animal studies have found that some polyphenols have the ability to protect the skin from the damaging effects of UV radiation, including a reduction in skin inflammation, oxidative stress, and DNA damage [57]. The compounds studied cover a wide range, including green tea polyphenols, grape seed proanthocyanidins, resveratrol, and genistein.

While some of these phytonutrients may be isolated and serve as the subjects of further study in the future, it is important to take into account the role of synergy. Multiple phytochemicals have demonstrated synergistic effects, as has been documented in both laboratory and animal studies. In a study of reconstituted human serum, beta-carotene, vitamin C, and vitamin E provided synergistic protection against oxidation [58]. In a study of murine skin, researchers administered various combinations of phytochemicals including topical resveratrol and oral ellagic acid, calcium D-glucarate, and grape seed extract [59]. Different combinations acted as potent inhibitors of skin tumorigenesis, and all combinations showed either additive or synergistic effects.

It is well recognized that there is a complex interplay of nutrients present in naturally occurring foods. In consuming a diet based on whole foods, the finely balanced proportion of nutrients, the large number of potentially protective compounds, and the other plant constituents (such as fiber) may all be necessary. Some compounds may potentiate the effects of others, and the role of synergy may make the whole more powerful than the sum of its parts. While there has historically been a focus on the effects of isolated nutrients in human subjects, it is just as vital, if not more so, to continue to study the effects of the entire package of interacting nutrients and substances found in whole foods.

## 9. Future Study Design 

Studies performed to date on diet and nonmelanoma skin cancer have had notable limitations, including small sample sizes in some as well as the research methodology challenges that arise in any study of diet and cancer. To begin with, almost all studies that examine the link between diet and cancer are based on data provided by the patients. Researchers determine nutrient intake based on food frequency questionnaires (FFQ). Participants are asked to estimate their food intake over a specified time period. For example, participants may be given a FFQ which requires estimating their intake of 129 food groups over timespans of 6 months [60]. It is well-recognized that there are issues with the reliability and validity of FFQs. As one research group has stated, “Researchers now recognize that data from FFQs and other dietary assessment methods can have substantial measurement errors, both systematic and random, which may lead to biased disease risk estimates [61].”

Another important issue regarding dietary interventions is that of timing. Given that sunburns in childhood can influence the development of skin cancer decades later, at what point in this process are we able to successfully intervene? The duration of dietary changes necessary to impact change is not known either. Is a 1-year dietary change sufficient, or would a decade of change be necessary?

While larger studies are certainly warranted, they entail other challenges. In one RCT described earlier, over 48,000 women were randomly assigned to a control diet or a study diet [44]. Participants in the intervention arm were given a dietary plan which entailed decreasing fat intake to less than 20% of caloric intake and consuming at least 5 servings of fruits and vegetables daily. While mandatory nutritional counseling sessions were provided, only 57% of individuals in the study arm were compliant with attendance at 3 years, and only 31% were compliant at 6 years (in contrast, the control group had compliance rates of 87% and 75%). To assess dietary intake, the FFQ was used at baseline, year one, and then every three years. These limitations must be considered when evaluating these results.

Further research is warranted, and lessons learned from the DASH trial may help in future dietary intervention trials. In designing the study, researchers blindly and randomly assigned participants to one of three diets. During an initial three-week phase, participants were studied to see if they would be fully compliant with the study design. Compliant participants then advanced to the intervention portion of the study, in which they ate one meal daily at the study center. Other meals were prepared for them to eat at home.

Adherence, reported as greater than 95% for all groups, was assessed through attendance at daily meals, daily diet diaries, and 24-hour urinary studies to verify dietary compliance. While this hypertension study was only 8 weeks in duration, it does serve as an example of a well-controlled dietary intervention in humans [52, 53, 62].

## 10. Conclusion

Dietary AOs may prevent free radical-mediated DNA damage and tumorigenesis secondary to UV radiation. Numerous laboratory studies have found that certain dietary AOs show significant promise in skin cancer prevention. These results have been substantiated by animal studies.

In human studies, researchers have evaluated both oral AO supplements and dietary intake of AOs via whole foods. Large RCTs of 4 specific AO supplements have been performed and have not shown efficacy. At this point in time these supplements are not recommended for NMSC prevention. Evaluation of other AO supplements, or AO combination supplements, may be warranted in the future based on laboratory and animal studies. Even this approach must be taken with caution, as research has already shown that some combinations of AOs may be linked to increased cancer risk. For example, the SU.VI.MAX trial found an increased risk of certain cancers in some subpopulations taking an AO combination supplement, specifically an increased risk of melanoma in supplemented women and an increased incidence of prostate cancer in men with an elevated prostate specific antigen at baseline [63].

In contrast, the results of human studies have indicated some promising trends when dietary AOs are provided via whole food intake. Regardless of the scientific interest in supplements, it is imperative that future studies evaluate the effects of dietary AOs provided via intake of whole foods. This is a vital area of research, especially given what we know of the mechanisms of oxidation, the evidence of benefit from many other phytonutrients in food, and the evidence of food synergy. Further research is warranted, despite the well-known challenges of studying whole food dietary interventions. In the meantime, there are many other reasons to recommend a diet strong in AOs, primarily the lack of side effects and known utility in the prevention of other cancers.

## Figures and Tables

**Table 1:** Experimental studies of AO supplements and NMSC incidence in human subjects.

Study	Study design	Antioxidant and other supplements studied	Effect on NMSC risk	Statistically significant results	Study location
Frieling et al., 2000 [35]	RCT of 22,071 male physicians over 12 years	50 mg beta-carotene, QOD	None	NA	USA
					
Duffield-Lillico et al., 2003 [36]	RCT of 1312 patients with previous NMSC over 10 years	200 mcg selenium, QD	BCC: none SCC: increased risk	BCC: NA SCC: RR: 1.14; 95% CI, 0.93–1.39	USA
					
Reid et al., 2008 [37]	RCT of 424 adults followed for 6 years, and a subset of 315 adults in the NPC study [37]	200 mcg selenium [NPC subset], QD	NMSC: increased risk	200 mcg cohort: RR: 1.49; 95% CI: 1.10–2.03; *P*: 0.008	USA
		400 mcg selenium, QD	None	NA	
					
Greenberg et al., 1990 [38]	RCT of 1805 patients with history of NMSC over 5 years	50 mg beta-carotene, QD	None	RR: 1.05; 95% CI, 0.91–1.22	USA
					
Hercberg et al., 2007 [39]	RCT of 13,017 adults followed over median of 7.5 years	Daily combination of: 120 mg vitamin C 30 mg vitamin E 6 mg beta-carotene 100 mcg selenium 20 mg zinc	BCC: none SCC: increased risk in women	BCC: NA SCC: Women: aHR: 1.68; *P*: 0.03	France
					
Vinceti et al., 2014 [40]	Meta-analysis which included 3 RCTs of NMSC	Selenium^+^	NMSC: increased risk	RR: 1.44; 95% CI: 0.95–1.17	Asia, Europe, US, and Australia
					
Chang et al., 2011 [41]	Meta-analysis of 10 RCTs	Vitamin A^+^ Vitamin C^+^ Vitamin E^+^ B-carotene^+^	None	NA	USA, Netherlands, Australia, UK, and Canada

^+^Dosages varied. aHR: adjusted hazard ratio; BCC: basal cell carcinoma; CI: confidence interval; NA: not applicable; NMSC: nonmelanoma skin cancer; mg: milligrams; mcg: micrograms; QD: daily dosing; QOD: every other day dosing; *P*: *P* value; RCT: randomized control trial. RR: relative rate; SCC: squamous cell carcinoma.

**Table 2:** Observational studies of dietary intake via supplements combined with food and NMSC incidence.

Study	Study design	Method of assessing dietary intake	Antioxidant and other supplements studied	Effect on NMSC risk	Statistically significant results	Study location
van Dam et al., 2000, Health Professionals Follow-Up Study (HPFS) [64]	Prospective cohort study of 3,217 males followed for 8 years	FFQs	Retinol Vitamin C Vitamin E	BCC: no reduction	NA	USA
						
Fung et al., 2002, Nurses Health Study (NHS) [65]	Prospective cohort study of 85,836 women followed for 12 years	FFQs	Vitamin A Vitamin C Vitamin E B-carotene	BCC: no reduction; weakly positive trends seen	NA	USA
						
Fung et al., 2003 [66]	Prospective cohort study of HPFS and NHS followed women for 14 years and men for 10 years	FFQs	Retinol Vitamin A Vitamin C Vitamin E Carotenoids	SCC: no reduction	NA	USA

BCC: basal cell carcinoma; FFQs: food frequency questionnaires; NA: not applicable; NMSC: nonmelanoma skin cancer; SCC: squamous cell carcinoma.

**Table 3:** Serum AO levels and NMSC incidence.

Study	Study design	Antioxidant supplements studied	Effect on NMSC risk	Statistically significant results	Study location
Clark et al., 1984 [42]	Case-control study of 240 subjects with NMSC	Selenium	Significantly lower levels in patients with NMSC	BCC: OR: 3.91; 95% CI: 1.2–13.1	USA
					
Breslow et al., 1995 [67]	Nested case-control study of 30 BCC and 37 SCC patients using serum obtained prior to diagnosis of NMSC	Retinol Beta-carotene *α*-Tocopherol Selenium	None	NA	USA
					
Dorgan et al., 2004 [68]	Prospective cohort study of 302 subjects with history of BCC followed for 5 years	Carotenoids Vitamin E	None	NA	USA
					
		Carotenoids	None	NA	
van der Pols et al., 2009 [69]	Prospective cohort study of 485 Australian adults followed for 8 years	Selenium	60% decreased incidence high serum levels	BCC: RR: 0.43; 95% CI: 0.21–0.86; *P*: 0.02 SCC: RR: 0.36; 85% CI: 0.15–0.82; *P*: 0.02	Australia
		Vitamin E	None	NA	

BCC: basal cell carcinoma; CI: confidence interval; NA: not applicable; NMSC: nonmelanoma skin cancer; OR: odds ratio; *P*: *P* value; RR: relative rate; SCC: squamous cell carcinoma.

**Table 4:** AO intake via whole foods and NMSC incidence.

Study	Study design	Method of assessing dietary intake	Diet(s) studied	Effect on NMSC risk	Reported statistical results	Study location
*Observational studies*						
Ibiebele et al., 2007^a^ [45]	Prospective observational study of 1360 adults followed over the course of 11 years	FFQs every 6 months	Meat & fat pattern	SCC: increased risk	RR: 3.77; 95% CI: 1.65–8.63; *P*: 0.002^b^	Australia
			Vegetable & fruit pattern	SCC: decreased risk by 54%^c^	RR: 0.46; 95% CI: 0.23–0.91; *P*: 0.02^b,c^	
						
van der Pols et al., 2011^a^ [60]	Prospective observational study of 1056 Australian adults over the course of 11 years	FFQs every 6 months	129 different food groups	None	No significant findings among food groups	Australia

*Experimental studies*						
Black et al., 1995 [70]	Randomized control trial of 101 skin cancer patients followed over the course of 2 years	Complete daily food records	Low fat, high in beta-carotene, vitamin C, and fiber	Significant reduction	*P* < 0.05	USA
						
Gamba et al., 2013 [44]	Randomized control trial of over 48,000 postmenopausal women followed over the course of 8 years	FFQs at baseline, year one, and then every 3 years	Low fat, high in fruits, vegetables, and grains	None	NA	USA

^a^Used data from the ongoing Nambour Skin Cancer Study [71]. ^b^Reported between lowest and highest tertiles of antioxidant intake. ^c^In individuals with a history of skin cancer. BCC: basal cell carcinoma; CI: confidence interval; FFQs: food frequency questionnaires; NA: not applicable; NMSC: nonmelanoma skin cancer; OR: odds ratio; *P*: *P* value; RR: relative rate; SCC: squamous cell carcinoma.
